# Impact of Coffee Consumption on Fructose-Related Metabolic Alterations: Potential Mechanisms and Implications for Metabolic Diseases

**DOI:** 10.3390/diseases14080288

**Published:** 2026-08-11

**Authors:** Alejandro Castañeda-López, Fernando Suárez-Sánchez, Fengyang Huang, Miguel Cruz, Adrián Hernández-Díazcouder

**Affiliations:** 1 Unidad de Investigación Médica en Bioquímica, Hospital de Especialidades, Centro Médico Nacional Siglo XXI, Instituto Mexicano del Seguro Social (IMSS), Mexico City 06720, Mexico; alex.qfb9@gmail.com (A.C.-L.);; 2 Laboratorio de Investigación en Obesidad y Asma, Hospital Infantil de México Federico Gómez, Mexico City 06720, Mexico

**Keywords:** sugar-sweetened beverages, insulin resistance, gut microbiota, polyphenols, metabolic dysfunction-associated steatotic liver disease, oxidative stress

## Abstract

Coffee is one of the most widely consumed beverages worldwide. Coffee and its bioactive compounds, including caffeine, chlorogenic acid, and caffeic acid, have attracted increasing attention because of their potential health benefits. Evidence from pediatric and adult populations supports a positive association between fructose intake from sugar-sweetened beverages and the increasing prevalence of obesity and other non-communicable diseases. In this context, coffee consumption may represent a potential protective dietary factor against high fructose intake-induced metabolic alterations, including obesity, type 2 diabetes, liver disease, cardiovascular disease, and alterations in gut microbiota composition. Therefore, this review summarizes current evidence on the potential role of coffee consumption and coffee-derived bioactive compounds in modulating fructose-induced metabolic alterations and discusses the mechanisms involved. However, current evidence from human studies remains limited, and the clinical relevance of the beneficial effects observed in experimental models requires confirmation through well-designed clinical trials.

## 1. Introduction

Coffee is one of the most widely consumed beverages worldwide and has been valued since ancient times for its stimulant properties and distinctive flavor. Its chemical composition is highly complex, comprising more than 1000 phytochemicals, including methylxanthines, amino acids, phenolic acids, and other polyphenols [1]. This composition varies according to species, bean quality, roasting degree, and brewing method [1]. Roasting progressively reduces chlorogenic acid content through thermal degradation, leading to the formation of caffeic acid, chlorogenic acid lactones, and other phenolic derivatives. In addition, brewing conditions, including water temperature, extraction time, and grind size, further influence the final concentration of these bioactive compounds in the beverage [2]. Roasting may reduce antioxidant polyphenols, whereas caffeine content is generally higher in Robusta coffee (*Coffea canephora*) than in Arabica coffee (*Coffea arabica* L.) [1]. Based on the brewing process, coffee can be broadly classified as boiled unfiltered, filtered, or decaffeinated coffee, with its final chemical profile influenced by grind size, water-to-coffee ratio, temperature, and extraction time [3].

Coffee consumption varies markedly across countries in terms of per-capita intake, total volume, and preferred coffee type, suggesting that exposure to coffee-derived bioactive compounds may differ according to population and consumption patterns [4,5].

In recent years, coffee and its bioactive compounds, including caffeine, chlorogenic acid (CGA), and caffeic acid, have received increasing attention due to their potential contribution to the health benefits associated with coffee intake [6]. In this context, coffee consumption may represent a potential protective dietary factor against metabolic alterations promoted by Western dietary patterns, particularly those associated with high fructose intake.

Excessive fructose intake, mainly through sugar-sweetened beverages (SSBs), has emerged as an important dietary factor associated with metabolic disturbances, including insulin resistance (IR), dyslipidemia, hepatic lipid accumulation, oxidative stress, and low-grade inflammation. Globally, high SSB consumption has increased substantially over the past three decades, particularly among young adults, and remains highly prevalent, especially in high-income countries and Latin America and the Caribbean [7,8]. In Mexico, SSBs are the most frequently consumed beverage category across all age groups, highlighting their widespread contribution to sugar intake [8]. Moreover, global SSB consumption is projected to continue increasing through 2050, underscoring the growing public health relevance of fructose-related metabolic risk [8].

This review aims to summarize current evidence on whether coffee consumption and coffee-derived bioactive compounds can modulate fructose-induced metabolic alterations and discuss the potential mechanisms involved.

Because direct evidence evaluating the interaction between coffee consumption and fructose-induced metabolic alterations remains limited, much of the available literature has independently examined the metabolic effects of fructose intake and coffee consumption. Therefore, Section 2 and Section 4 provide the necessary background on each topic, whereas Section 5 focuses on the available direct evidence evaluating coffee under fructose-related conditions. Currently, this evidence consists primarily of animal studies, with only one human intervention study identified. While these studies provide valuable mechanistic insights, the limited availability of well-designed human studies precludes definitive conclusions regarding the clinical relevance of these findings. In addition, much of the available human evidence is derived from observational studies, which cannot establish causal relationships and may be influenced by residual confounding, particularly smoking, dietary patterns, physical activity, and other lifestyle-related factors. Moreover, the biological effects of coffee may vary substantially across studies because coffee is a heterogeneous dietary exposure. Differences in coffee species, roasting degree, brewing method, bioactive compound composition, serving size, habitual intake, and the use of additives (e.g., sugar, artificial sweeteners, or milk) may contribute to variability in the reported metabolic outcomes. These methodological and biological considerations should therefore be considered when interpreting and comparing findings across studies.

The literature included in this narrative review was identified through searches of PubMed and Google Scholar conducted up to May 2026. The main search terms included “coffee consumption” AND fructose, “coffee consumption” AND metabolic alterations, “fructose” AND metabolic alterations, and “coffee-derived bioactive compounds” AND fructose. Additional relevant articles were identified by screening the reference lists of selected publications. Original research articles, clinical studies, experimental animal studies, and relevant review articles published in English were included based on their relevance to the objectives of this review.

## 2. Fructose Intake from Sugar-Sweetened Beverages and Metabolic Disease Risk

Evidence from pediatric and adult populations supports a positive association between fructose intake from SSBs and the increasing prevalence of obesity and other non-communicable diseases, with the magnitude of these effects depending on the amount, frequency, and duration of exposure [9]. In children and adolescents, higher SSB consumption has been associated with an increased risk of incident metabolic syndrome, abdominal obesity, and hypertension [10]. Similar associations have been reported in adults, in whom higher SSB intake has been linked to elevated diastolic blood pressure, body mass index, triglycerides, fasting glucose, cardiovascular mortality, and metabolic dysfunction-associated steatotic liver disease (MASLD) [11,12,13].

Evidence from intervention studies further supports these metabolic effects. Controlled trials have shown that fructose-sweetened beverages impair hepatic insulin sensitivity, increase circulating LDL-C, total cholesterol, and postprandial triglyceride concentrations, and promote hepatic de novo lipogenesis compared with glucose-sweetened beverages [14,15,16]. In contrast, one study reported that high-fructose intake did not alter ectopic lipid deposition or cardiac structure and function in healthy individuals, although these alterations were evident in insulin-resistant patients with non-alcoholic fatty liver disease (NAFLD), suggesting that the metabolic effects of fructose may depend on the underlying metabolic status [17].

In addition to hepatic and metabolic effects, high fructose intake may also influence metabolic risk through changes in the gut microbiota. In healthy women, a pilot study showed that a fruit-rich diet providing 100 g/day of fructose increased Firmicutes and butyrate-producing bacteria, whereas a high-fructose syrup diet reduced beneficial taxa such as *Faecalibacterium* and *Erysipelatoclostridium* [18]. However, these findings are not consistent across studies and may depend on the fructose source, fiber content, and food matrix [19,20]. In murine models, fructose-rich diets have been associated with intestinal dysbiosis, including reduced *Bacteroidetes*, increased *Proteobacteria*, and changes in taxa related to short-chain fatty acid (SCFA) production, intestinal barrier integrity, and inflammatory responses [21,22,23]. Therefore, alterations in the gut microbiota may be another mechanism by which high fructose intake contributes to metabolic alterations.

Taken together, current evidence supports fructose intake from SSBs as an important dietary contributor to obesity, metabolic alterations, liver disease, and cardiovascular risk.

## 3. Coffee Consumption

According to consolidated data from the International Coffee Organization, global coffee consumption is projected to reach a record 169.4–172.5 million 60-kg bags, equivalent to an approximate daily intake of 2.25 billion cups worldwide [4]. Northern Europe has the highest density of chronic coffee exposure. Finland ranks as the leading country in per-capita consumption, with an estimated per-capita consumption of approximately 12 kg/person/year. In contrast, the United States represents the largest critical mass in terms of total volume, consuming 1.43 million tons annually, while 66% of adults report daily coffee intake [5].

Although Mexico ranks among the world’s leading coffee-producing countries, its domestic per-capita consumption remains moderate to low compared with that of major importing nations. Average individual intake is estimated at only 0.3 cups/day (approximately 5 cups/week), corresponding to an annual per-capita consumption of roughly 1.3–1.7 kg/person/year [24]. Nevertheless, United States Department of Agriculture reports indicate that total domestic consumption reaches approximately 3.9 million 60-kg bags annually, underscoring the substantial size of the internal market despite relatively low individual intake [24]. In addition, instant coffee continues to dominate the Mexican market, accounting for 57% of total domestic consumption, whereas ground coffee represents the remaining 43% [24]. These differences in coffee consumption patterns are relevant because coffee type, preparation method, and additive use may influence the metabolic effects associated with coffee intake.

## 4. Coffee Intake and Metabolic Disease-Related Outcomes

### 4.1. Obesity

Emerging evidence suggests that the association between coffee consumption and obesity-related outcomes is heterogeneous (Table 1). A cross-sectional study including 1855 participants found that moderate coffee intake (2–3 cups/day) was associated with a more favorable body composition and inflammatory profile. Similar favorable associations were observed among individuals who consumed unsweetened coffee compared with those who consumed sweetened coffee, suggesting that added sugar may attenuate the potential metabolic benefits associated with coffee consumption [25]. This attenuation may be explained by the additional fructose and glycemic load provided by sweeteners, which can promote hepatic de novo lipogenesis, oxidative stress, insulin resistance, and low-grade inflammation, thereby potentially counteracting the antioxidant and insulin-sensitizing properties of coffee-derived bioactive compounds. Moreover, the addition of sugar increases the overall caloric content of coffee, which may further contribute to adverse metabolic outcomes. However, the study did not distinguish coffee subtypes or brewing methods, limiting the ability to determine whether the observed associations vary according to coffee preparation. Moreover, a randomized crossover interventional study including 38 participants with overweight or obesity found that the consumption of 3 cups/day of both roasted and lightly roasted coffee for 12 weeks reduced fat mass and body fat percentage, while slightly increasing muscle mass percentage. Notably, the reduction in body fat percentage was greater after the consumption of lightly roasted coffee than after roasted coffee [26]. Conversely, a prospective study with a 2-year follow-up in 170 kidney transplant recipients showed that compared with lower coffee consumption (0–1 times/day), higher intake (2–3 times/day) was associated with greater waist circumference, higher waist-to-height ratio, increased odds of central obesity, and poorer muscle quality [27]. Overall, the available evidence suggests that the health effects of coffee consumption are not uniform and may vary according to underlying health status.

In addition, studies suggest that the association between coffee consumption and adiposity may vary according to sex. A meta-analysis of 12 epidemiologic studies found that higher coffee intake (0 cups/day vs. ≥3 cups/day) was modestly associated with lower BMI and waist circumference, particularly in men [28]. Another meta-analysis of 23 studies reported a significant positive association between coffee consumption (≥1 to >6 cups/day) and the odds of general obesity in women [29]. Individual studies further support these sex-specific differences, showing that light coffee intake (one cup/day) was associated with lower odds of sarcopenia in men, whereas more frequent coffee consumption was linked to higher odds of both general and central obesity in women [30]. This is particularly relevant because obesity is one of the main metabolic outcomes associated with chronic high-fructose intake.

### 4.2. Type 2 Diabetes

Accumulating evidence suggests that coffee consumption may be inversely associated with the risk of type 2 diabetes (T2D), although the magnitude of this association may vary according to coffee composition, additive use, and population characteristics (Table 2). In a cohort study including 334 individuals with prediabetes followed for 3 years, coffee consumption was associated with a nearly twofold higher likelihood of returning to normoglycemia [31]. In addition, compared with non-drinkers, coffee drinkers showed significantly lower 2-h serum glucose concentrations over time [31]. A meta-analysis including 15 studies found that sugar-sweetened beverage consumption was associated with a higher risk of both incident type 2 diabetes and mortality, whereas coffee intake was associated with a lower risk of T2D incidence and mortality [32]. Moreover, in three large prospective cohorts comprising 3,665,408 person-years of follow-up, each additional cup of coffee consumed without additives was associated with a 10% lower risk of T2D. However, the addition of sugar or artificial sweeteners significantly attenuated this inverse association, suggesting that these additives may weaken the protective association of coffee with T2D risk [33]. Furthermore, evidence from two large prospective cohorts with median follow-ups of 13.9 and 7.4 years showed that each 1-cup/day increase in coffee consumption was associated with a 4% lower risk of T2D. Higher coffee intake was also associated with lower homeostatic Model Assessment for Insulin Resistance and C-reactive protein levels, as well as higher adiponectin and IL-13 concentrations and lower leptin levels, which may partly explain this inverse association [34]. Consistently, a dose–response meta-analysis of 12 cohort studies including 266,183 men found that each additional daily cup of coffee was associated with a lower risk of T2D, although the association appeared to be nonlinear, with moderate intake appearing to confer greater benefit than higher consumption levels [35]. In a cross-sectional study including 6311 women with a median age of 53 years, higher intake of caffeinated coffee (>3 cups/day) was associated with a lower risk of T2D, but not with gestational diabetes mellitus [36]. Notably, this inverse association appeared to be more pronounced among individuals with obesity and those with lower levels of physical activity [36]. Overall, current evidence supports an inverse association between coffee consumption and T2D risk, with moderate intake and coffee consumed without additives appearing to show the most consistent favorable associations. However, most of the available evidence is derived from observational studies, which cannot establish causal relationships, and additional well-designed randomized clinical trials are needed to confirm these findings. These associations are relevant in the context of fructose-induced insulin resistance, one of the central mechanisms linking SSB intake with metabolic disease.

### 4.3. Liver Diseases and MASLD

Because MASLD has recently replaced NAFLD in the updated nomenclature, both terms are used according to the terminology applied in the cited studies. Accumulating evidence suggests that the association between coffee consumption and liver-related outcomes may vary according to coffee type, sweetener use, and the specific hepatic endpoint evaluated (Table 3). In a longitudinal cohort of 170,044 participants followed for a median of 12.4 years, unsweetened coffee consumption (>3.5 cups/day) was associated with the lowest risk of chronic liver disease and liver-related events, consistent with a potential protective association [37]. In contrast, sweetened coffee showed less consistent associations, while added sugar or artificial sweeteners were linked to a higher risk of adverse liver outcomes, particularly at higher artificial sweetener intake [37]. Similarly, in a cross-sectional study of 8062 participants, coffee consumption was inversely associated with NAFLD risk, with a 21% lower risk among those consuming > 4 cups/day; this association appeared stronger in women and in individuals with lower educational attainment [38]. In addition, in a cohort study of 1079 participants with a median age of 59 years, higher coffee consumption was associated with a lower risk of developing metabolic dysfunction-associated steatotic liver disease (MASLD), with the greatest reduction observed among those consuming 4–6 cups/day (59.3%) [39]. Consistently, another cohort including 185,437 participants with a median follow-up of 10.49 years found that intake of >2.5 cups/day of unsweetened coffee, caffeinated coffee, or both was associated with a lower risk of MASLD, with the inverse association being particularly evident for caffeinated coffee [40]. Taken together, the available evidence suggests that unsweetened and caffeinated coffee may be more consistently associated with favorable liver-related outcomes than sweetened coffee or coffee with added sweeteners.

Conversely, a study including 5266 participants without MASLD and 1326 with MASLD but without advanced liver fibrosis found that coffee consumption was not associated with a lower risk of incident MASLD, but intake of ≥2 cups/day was linked to a lower risk of fibrosis progression in those with pre-existing MASLD, indicating that the potential benefits of coffee may be more evident during disease progression than disease onset [41]. Moreover, a meta-analysis of 14 randomized controlled trials including 897 participants found no significant effect of coffee consumption on liver enzymes, although it increased the adiponectin concentrations, which may indicate a potential benefit for liver dysfunction [42]. Overall, coffee consumption appears to be more consistently associated with a lower risk of adverse liver outcomes and fibrosis progression than with incident MASLD.

### 4.4. Cardiovascular Disease

Several studies have suggested a protective role of coffee consumption against cardiovascular disease (CVD) (Table 4). In a cohort study including 449,563 participants with a median age of 58 years and a median follow-up of 12.5 years, consumption of all coffee subtypes (decaffeinated, ground, and instant) was associated with a lower risk of incident CVD, with the lowest risk generally observed at 2–3 cups/day, particularly for decaffeinated and instant coffee [43]. In addition, consumption of both ground and instant coffee was associated with a lower risk of incident arrhythmia, with the greatest risk reduction observed at 4–5 cups/day for ground coffee [43]. Similarly, among never-smoking adults with T2D, moderate coffee consumption (2–4 cups/day) was associated with a lower risk of total CVD, coronary heart disease, myocardial infarction, and heart failure, consistent with a favorable association with cardiovascular health even in metabolically vulnerable populations. Notably, this inverse association with total CVD was observed for instant and ground coffee, but not for decaffeinated coffee, whereas the lower risk of major vascular disease was evident for decaffeinated and instant coffee, with a similar but non-significant trend for ground coffee [44]. In contrast, subgroup analyses from a meta-analysis of 32 prospective cohort studies suggested that coffee consumption was associated with an increased risk of coronary heart disease (CHD) in men, whereas a potentially reduced risk was observed in women [45]. The same meta-analysis also reported a higher risk of CHD in studies with follow-up periods of 20 years or longer [45]. Therefore, the available evidence suggests that the cardiovascular effects of coffee may vary according to the subtype consumed. However, further experimental and longitudinal studies are required to better define the specific impact of each coffee subtype and to elucidate the mechanisms underlying these associations.

In addition to its potential protective effects, coffee consumption has been associated with a lower risk of cardiovascular mortality. In a meta-analysis of ten prospective cohort studies including 82,270 participants with T2D, coffee consumption (4 cups/day) was associated with a lower risk of cardiovascular mortality, CHD mortality, CHD, and total cardiovascular events [46]. In a study including 10,639 participants, coffee consumption of ≥540 g/day was associated with a reduced risk of CVD mortality, and this risk decreased significantly with increasing coffee intake among coffee consumers, suggesting an inverse dose–response association [47]. In the same study, prolonged sedentary behavior was associated with increased all-cause mortality only among non-coffee drinkers, indicating that the harmful effect of sedentary time may be more pronounced in the absence of coffee consumption [47]. In contrast, a study including 11,697 women with a median age of 66 years found no association between filtered caffeinated coffee consumption or caffeine intake and cardiovascular mortality, indicating no clear beneficial or harmful effect of coffee in this population [48]. Taken together, the evidence suggests a potential protective role of coffee against CVD and cardiovascular mortality in some populations. However, the findings are not entirely consistent, as null or adverse associations have also been reported in specific subgroups and study settings.

### 4.5. Gut Microbiota Modulation and Dysbiosis-Related Taxa

Evidence suggests that individuals who consume moderate amounts of coffee (<4 cups/day) exhibit greater gut microbial diversity and a higher abundance of beneficial microorganisms when compared to non-consumers. Particularly, the abundance of *Bifidobacterium* spp. tends to consistently increase across studies, whereas the abundance of *Enterobacteriaceae* decreases [49,50]. *Bifidobacterium* species participate in the production of SCFAs, which has been associated with beneficial effects such as enhanced gut barrier function. *Bifidobacterium* can also inhibit pathogenic bacteria growth [51]. On the other hand, bacteria of the *Enterobacteriaceae* family have been associated with gut dysbiosis, inflammatory conditions, and the growth of opportunistic pathogens.

Coffee consumption has also been associated with increased abundance of *Lawsonibacter asaccharolyticus*, *Alistipes*, and *Faecalibacterium*. Notably, *L.* asaccharolyticus showed the strongest association with coffee consumption in a multi-cohort study, exhibiting a 4.5- to 8-fold higher abundance in coffee drinkers than in non-drinkers. Such an abundance increase was correlated with concentrations of quinic acid and its potential derivatives in plasma [52]. This compound can exert antioxidant properties by scavenging reactive oxygen species and reducing oxidative stress. Some evidence also suggests anti-inflammatory effects through the modulation of inflammatory signaling pathways [53]. *Faecalibacterium* is another genus whose abundance is related to coffee consumption. Particularly, *Faecalibacterium prausnitzii* is one of the main butyrate producers and its abundance in the gut has been associated with anti-inflammatory effects, gut health, and even proposed as a probiotic [54]. The role of *Alistipes* is more complex. Some studies suggest potential beneficial metabolic functions, but others have linked certain species to disease states such as depression, colorectal cancer, and inflammation [55]. In this context, bacterial species or strain present in the gut may become paramount.

Due to the capacity of coffee to produce changes in the abundance of some bacterial species associated with beneficial effects, it may be regarded as a potential prebiotic. Several coffee compounds can be metabolized by the gut bacteria, leading to the production of short-chain fatty acids such as butyrate, acetate, and propionate [56]. Especially, the production of butyrate is of particular importance because its concentration in the intestine has been associated with an enhanced intestinal barrier integrity and reduced inflammation. Acetate, on the other hand, can affect gene expression regulation by participating in histone acetylation and has also been recognized as an important immunomodulatory metabolite [57].

Despite the evidence linking coffee consumption to changes in the gut microbiota, the magnitude of the microbiota modifications and effects is not homogeneous across studies (Table 5). It may depend on factors such as baseline microbial composition, host genetics, overall dietary patterns, coffee preparation methods, and the use of additives such as sweeteners and milk.

## 5. Coffee and Coffee-Derived Bioactive Compounds Under Fructose-Related Conditions

Although direct clinical evidence evaluating coffee under fructose-related conditions remains limited, current evidence is limited to a single human intervention study, whereas most of the available mechanistic evidence derives from experimental animal models (Table 6). Nevertheless, the available evidence suggests that coffee consumption may partially modulate fructose-induced metabolic alterations. However, the clinical relevance of these findings remains uncertain and requires confirmation through well-designed human studies. In a randomized crossover trial in healthy young men, fructose overfeeding (4 g/kg/day for 6 days) increased intrahepatocellular lipid content and hepatic glucose production while reducing fasting lipid oxidation [58]. Under these conditions, intake of 4 cups/day of coffee attenuated the increase in hepatic glucose production, regardless of coffee type, which may reflect improved hepatic insulin sensitivity [58]. In addition, caffeinated coffee high in CGA and decaffeinated coffee with regular CGA content increased fasting lipid oxidation, whereas decaffeinated coffee high in CGA had no significant effect. None of the coffee preparations, however, prevented fructose-induced hepatic lipid accumulation [58]. Although experimental studies consistently suggest beneficial effects of coffee under fructose-rich conditions, clinical evidence remains scarce. This may, in part, reflect the inherent difficulty of conducting long-term dietary intervention trials evaluating coffee consumption under controlled high-fructose conditions. Such studies are costly, require sustained dietary adherence, and are particularly challenging in free-living populations, where fructose intake is difficult to standardize and monitor. Consequently, it remains uncertain whether the beneficial effects observed in animal models translate to humans. Well-designed clinical trials are therefore needed to clarify the role of coffee consumption under conditions of high fructose intake. Human equivalent doses were calculated using the body surface area normalization method proposed by Reagan-Shaw et al. [59] to facilitate the translation of animal doses to clinically relevant human equivalents.

In murine studies, coffee and coffee-derived preparations also showed beneficial metabolic effects under fructose-rich conditions. In rats fed a high-fat diet for 14 weeks, coffee and fructose provided in the same drinking bottle, corresponding to an approximate daily intake of 1.18 g of ground medium-roast coffee (equivalent to 4 cups of coffee per day), improved metabolic status by reducing body weight, TG, and plasma glucose, while also attenuating hepatic steatosis and IR [60]. Likewise, in rats fed a high-fructose diet, coffee intake at doses of 213 mg/kg body weight/day (equivalent to 3 cups per day) and 284 mg/kg body weight/day (equivalent to 4 cups per day) reduced body weight, fasting glucose, and uric acid levels, whereas the higher dose additionally decreased fasting TG and LDL-C concentrations [61]. Consistent with these findings, oral administration of an aqueous coffee parchment extract (100 mg/kg/day; equivalent to a human dose of 16.2 mg/kg/day) for 4 weeks in rats with high-fructose diet-induced obesity prevented adipose tissue accumulation and ameliorated hyperglycemia, hyperinsulinemia, hyperlipidemia, hepatic steatosis, and oxidative stress [62].

In an inflammatory setting, coffee intake also appeared to exert roast-dependent effects. In rats fed a high-fat/high-fructose diet, daily administration of 7.4 mL of coffee (equivalent to 75 mL/day in humans, which represents three standard cups (25 mL) of espresso in humans) for 16 weeks reduced hepatic nuclear factor kappa B (NF-κB) levels, with the strongest effect observed in the very dark roast group (approximately 75%), followed by the dark roast group (about 50%) and the unroasted coffee group (about 30%) [63]. A similar pattern was observed for adipose tissue tumor necrosis factor alpha (TNF-α), which decreased significantly in all coffee-treated groups, with the greatest reduction occurring in the very dark roast group [63].

In addition to coffee intake per se, coffee-derived extracts and isolated bioactive compounds have also shown favorable effects in murine models of high fructose intake. In obese rats fed a high-fructose diet, coffee extracts improved metabolic alterations, obesity-related biomarkers, and oxidative stress parameters, while green coffee also reduced hepatic interleukin (IL)-1β (IL-1β) and IL-6 levels, supporting an anti-inflammatory action [64]. Likewise, in rats fed a high-carbohydrate, high-fat diet with fructose, coffee extract improved glucose tolerance, reduced plasma insulin and systolic blood pressure, and attenuated cardiovascular and hepatic inflammation, fibrosis, and liver injury markers, despite having no effect on abdominal obesity [65]. These differences in dietary models should be considered when interpreting the magnitude and direction of the metabolic effects. Taken together, current evidence suggests that coffee, coffee-derived extracts, and isolated bioactive compounds may help alleviate metabolic and inflammatory disturbances under fructose-related conditions. In this context, growing evidence indicates that some of the beneficial effects of coffee on fructose-related alterations may be mediated by specific bioactive compounds, particularly caffeine, chlorogenic acid, and caffeic acid.

### 5.1. Caffeine

Available evidence suggests that caffeine may influence fructose-related metabolic responses in a context-dependent manner, with acute effects in humans and variable outcomes in experimental models. In healthy young and older adults, ingestion of 75 g fructose plus 300 mg caffeine elicited a greater increase in energy expenditure than glucose plus caffeine, whereas glucose and insulin responses remained lower after fructose than after glucose [66]. These findings suggest that caffeine may acutely influence the metabolic handling of fructose in humans.

**Figure 1:**
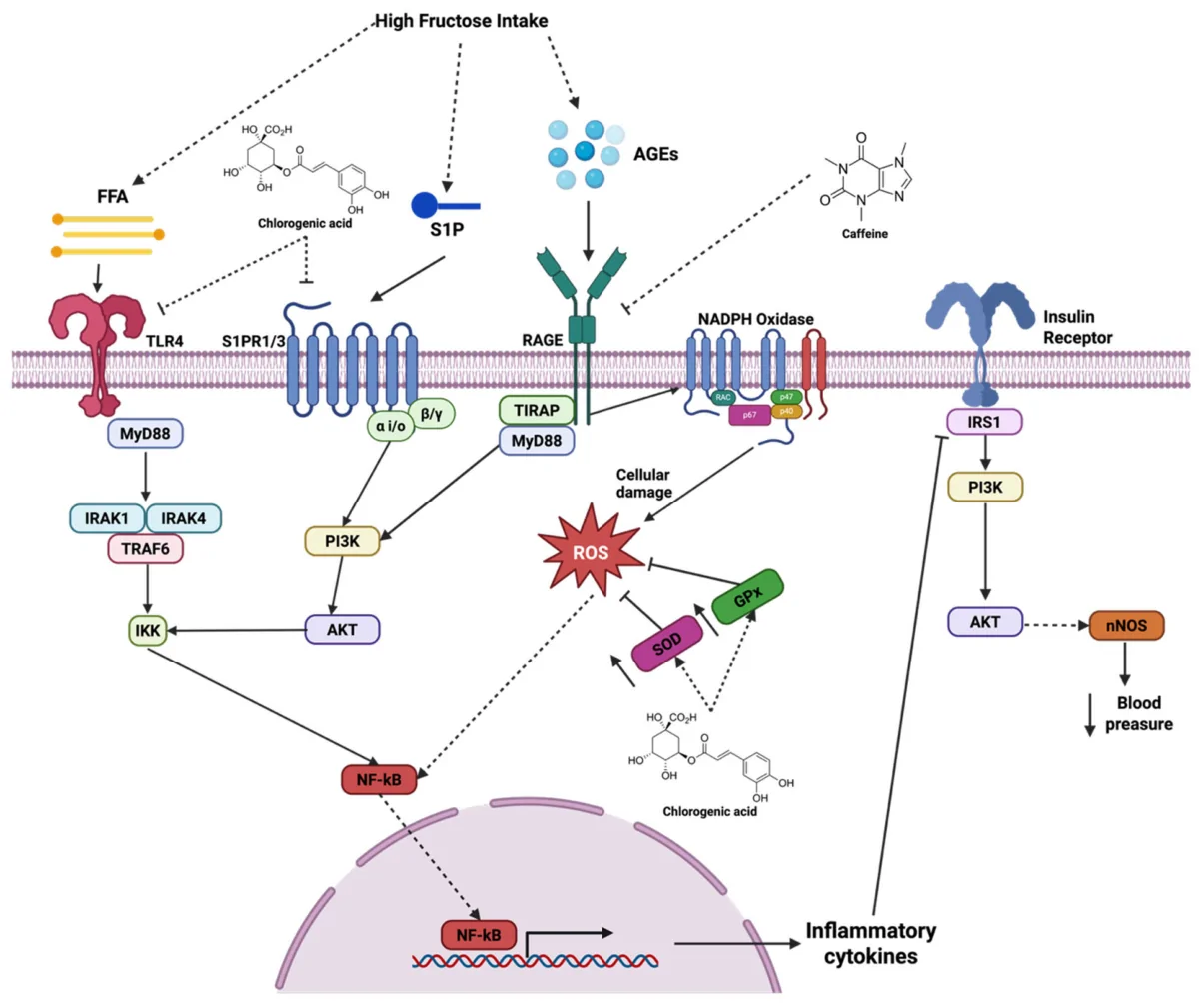
Proposed molecular mechanisms underlying the protective effects of coffee-derived bioactive compounds against fructose-induced metabolic alterations. High fructose intake may promote metabolic disturbances, including increased production of AGEs, activation of RAGE-mediated signaling, upregulation of the S1P/S1PR1/3 pathway, and lipid accumulation/FFA overload. These alterations may contribute to NADPH oxidase activation, increased ROS production, oxidative stress, inflammatory signaling, cellular damage, and impaired insulin signaling. Coffee-derived bioactive compounds, particularly caffeine and chlorogenic acid, may attenuate these effects by modulating RAGE/NADPH oxidase-dependent ROS production, enhancing antioxidant defenses through SOD and GPx, and reducing TLR4/NF-κB-mediated inflammatory responses. In addition, caffeine may improve central insulin signaling through the IRS1/PI3K/AKT/nNOS pathway, increasing NO bioavailability and contributing to blood pressure regulation. This figure summarizes the current evidence and presents an integrative conceptual model based on findings from separate experimental and clinical studies on fructose and coffee-derived bioactive compounds. It is intended to illustrate potential mechanisms and does not represent pathways validated within a single integrated experiment. AGEs: Advanced glycation end products; RAGE: Receptor for advanced glycation end products; FFA: Free fatty acid; NADPH: Nicotinamide adenine dinucleotide phosphate; ROS: Reactive oxygen species; SOD: Superoxide dismutase; GPx: Glutathione peroxidase; TLR4: Toll-like receptor 4; S1P: Sphingosine-1-phosphate; S1PR1/3: Sphingosine-1-phosphate receptor 1 and -3; NF-κB: Nuclear factor kappa B; NO: Nitric oxide; IRS1: Insulin receptor substrate 1; AKT: Protein kinase B; nNOS: Neuronal nitric oxide synthase.

In experimental models, caffeine administered at 15 mg/kg/day (equivalent to approximately 3–4 cups of coffee per day) for 4 weeks to rats fed a high-fructose diet improved metabolic status, including insulin sensitivity and blood pressure [67]. This effect may be related to enhanced insulin sensitivity in the nucleus tractus solitarii through reduced receptor for advanced glycation end product (RAGE)-mediated superoxide generation and increased nitric oxide (NO) production via the upregulation of insulin receptor substrate 1 (IRS1)–phosphoinositide 3-kinase (PI3K)–Akt–nNOS signaling (Figure 1) [67]. Given that fructose induces metabolic alterations, at least in part, through the regulation of RAGE expression in insulin-sensitive tissues [68,69,70], the beneficial effects of caffeine may involve the modulation of this pathway. In contrast, in rats fed a high-fat diet plus fructose, low-dose caffeine (5 mg/kg/day; equivalent to approximately one cup of coffee per day) did not reverse hepatic steatosis or improve insulin sensitivity. Moreover, caffeine increased hepatic stearoyl-CoA desaturase 1 expression, suggesting a limited effect on lipogenic pathways despite attenuating the increase in Cer 18:1 [71]. These findings suggest that caffeine may exert context-dependent effects, with potential benefits on insulin signaling but limited effects on hepatic lipid accumulation.

### 5.2. Chlorogenic Acid

Among the coffee-derived compounds, chlorogenic acid (CGA) appears to exert particularly relevant hepatoprotective and anti-inflammatory effects under fructose-rich conditions. In a model of fructose-induced NAFLD, CGA administered at 40 mg/kg/day (equivalent to 1–3 cups of coffee per day) for 8 weeks reduced serum lipids, glucose, IR, and liver injury markers, while increasing hepatic glutathione peroxidase (GPx) and superoxide dismutase (SOD) activity. These effects were accompanied by attenuation of hepatic inflammation, including lower IκB kinase (IKK), TNF-α, toll-like receptor 4 (TLR4), and NF-κB signaling, as well as suppression of the sphingosine kinase 1 (SphK1)/sphingosine-1-phosphate (S1P) pathway [72]. Since fructose has been shown to activate SphK1/S1P/sphingosine-1-phosphate receptor 1 (S1PR1)/3 signaling in liver and kidney tissues [73,74], CGA may exert part of its hepatoprotective effect by modulating this fructose-induced proinflammatory axis (Figure 1). Moreover, in rats fed a high-fat and high-fructose diet, dietary CGA at 2.6 mmol/kg (equivalent to 1–2 cup of coffee per day) reduced fasting glucose, total cholesterol, hepatic TG accumulation, alanine aminotransferase (ALT) and alkaline phosphatase (ALP) activities, and proinflammatory markers, while increasing adiponectin levels, despite not significantly affecting body weight or adiposity [75]. Taken together, the available evidence suggests that CGA improves the metabolic and inflammatory alterations induced by high fructose intake. However, further studies are needed to clarify the cellular mechanisms underlying its beneficial effects in this context.

### 5.3. Caffeic Acid

Caffeic acid has also shown consistent beneficial effects in experimental models of high fructose intake. Caffeic acid administered at 40 mg/kg body weight (human-equivalent dose of approximately 6.5 mg/kg/day, or about 450 mg/day) exerted metabolic, anti-inflammatory, and antioxidant effects in rats with high-fructose diet-induced metabolic syndrome. Treatment improved hyperglycemia, IR, dyslipidemia, and adipokine imbalance, while reducing the TNF-α, IL-6, and IL-8 levels [76]. In addition, caffeic acid restored hepatic antioxidant enzyme activities and attenuated oxidative damage markers, supporting its protective effect against fructose-induced metabolic disturbances [76]. Similarly, in rats fed a high-fat and high-fructose diet, caffeic acid supplied at an equimolar concentration of 2.6 mmol/kg diet (equivalent to a human dose of approximately 2.3 mg/kg/day, or about 161 mg/day) showed potential hepatoprotective and anti-inflammatory effects [75]. Treatment improved fasting glucose and total cholesterol, reduced hepatic TG accumulation, lowered serum ALT and ALP activities, and attenuated inflammatory markers, including IL-6, IL-1β, and C-reactive protein, while increasing the adiponectin levels [75]. These findings suggest that caffeic acid may contribute to the protective effects of coffee-derived compounds against fructose-induced metabolic dysfunction.

Although the available studies consistently suggest beneficial effects of coffee and its bioactive compounds under fructose-related conditions, these findings should be interpreted with caution because the evidence is derived predominantly from experimental animal models. Only one controlled human intervention study has directly evaluated this interaction. Therefore, the translational relevance of these mechanisms remains uncertain and requires confirmation in well-designed clinical trials.

## 6. Future Directions

Future studies should assess the effects of coffee and coffee-derived bioactive compounds on the regulation of non-coding RNAs, including microRNAs and long non-coding RNAs, associated with metabolic alterations induced by chronic fructose intake. In addition, the molecular mechanisms underlying the effects of coffee should be explored in metabolically relevant tissues beyond the liver, particularly adipose tissue and skeletal muscle. Further research is also needed to determine whether coffee intake modulates key pathways involved in fructose-induced metabolic alterations, including oxidative stress, inflammation, insulin signaling, lipid metabolism, mitochondrial function, and gut microbiota-derived signaling. Another important area of investigation is the potential modulation of endocrine-like communication mediated by extracellular vesicle-derived non-coding RNAs and its contribution to the systemic metabolic effects of coffee. Finally, well-controlled clinical trials are required to determine whether coffee intake can attenuate fructose-induced metabolic alterations in humans while considering coffee type, dose, roasting degree, brewing method, and the use of additives.

## 7. Conclusions

Current evidence suggests that coffee consumption and coffee-derived bioactive compounds may attenuate fructose-induced metabolic alterations, including insulin resistance, hepatic lipid accumulation, oxidative stress, inflammation, cardiovascular risk, and gut microbiota dysbiosis. These effects may be mediated by caffeine, chlorogenic acid, and caffeic acid through the regulation of glucose and lipid metabolism, inflammatory signaling, antioxidant defenses, and gut microbiota composition. Importantly, this review integrates the available evidence on the potential role of coffee and its bioactive compounds in counteracting fructose-induced metabolic alterations, providing a comprehensive overview of the underlying mechanisms and highlighting current knowledge gaps. Although epidemiological studies have consistently reported beneficial associations between coffee consumption and metabolic health, these findings should be interpreted with caution because most evidence is derived from observational studies, which remain susceptible to residual confounding and reverse causation despite adjustment for major lifestyle and socioeconomic factors. However, human evidence remains limited and heterogeneous. Therefore, well-designed randomized controlled trials are needed to determine whether the protective effects observed in experimental models translate to humans and to establish the causal role of coffee and its bioactive compounds in mitigating fructose-induced metabolic alterations.

## Figures and Tables

**Table 1:** Summary of human studies evaluating the association between coffee consumption and obesity.

Study Design	Population	Coffee Dose	Finding	Reference
Cross-sectional	1855 participants	2–3 cups/day	Lower BMI. Lower inflammatory profile.	[24]
Crossover, randomized, controlled, and single-blind intervention	38 participants with OW or Ob	3 cups/day for 12 weeks	Reduces fat mass and body fat percentage. Increase muscle mass.	[25]
Prospective study with a 2-year follow-up	170 kidney transplant recipients	2–3 cups/day	Greater waist circumference. Higher waist-to-height ratio. Increased odds of central obesity. Poorer muscle quality.	[26]
Meta-analysis	General adult population (12 epidemiologic studies)	1 cup/day vs. ≥3 cups/day	Men: Lower BMI. Waist circumference. Women: No associations.	[27]
Meta-analysis	207,551 participants (23 epidemiological studies)	From ≥1 to >6 cups/day	Men: No association with obesity. Women: Increased odds of obesity.	[28]
Cross-sectional	6906 participants with Ob	From ≤1 (light) to ≥3 cups/day (frequent)	Men: Light coffee intake lower odds of sarcopenia. Women: Frequent coffee intake higher odds general and central obesity.	[29]

BMI: Body mass index; OW: Overweight; Ob: Obesity.

**Table 2:** Summary of human studies evaluating the association between coffee consumption and T2D.

Study Design	Population	Coffee Dose	Finding	Reference
Cohort with a 3-year follow up	334 participants with prediabetes	Habitual coffee consumption (median: 58.3 mL/week)	Higher likelihood of regression to normoglycemia. Compared to non-drinker: Lower 2-h serum glucose concentrations over time.	[30]
Meta-analysis	15 observational studies	Highest vs. lowest coffee intake (study-specific categories)	Lower incidence of T2D.	[31]
Three large prospective cohorts	3,665,408 person-years of follow-up	Each additional cup of coffee consumed	10% lower risk of T2D	[32]
Two large prospective cohorts with median follow-ups of 13.9 and 7.4 years	UK-Biobank (145,368 participants). Rotterdam Study (7111 participants)	Each 1-cup/day	4% lower risk of T2D. Lower HOMA-IR. Lower CPR. High adiponectin and IL-13.	[33]
Meta-analysis	266,183 men (12 cohort studies)	Each additional 1-cup/day	Lower risk of T2D.	[34]
Cross-sectional	6311 women	>3 cups/day	Lower risk of T2D. No associated with gestational diabetes mellitus.	[35]

T2D: Type 2 diabetes; HOMA-IR: Homeostatic Model Assessment of Insulin Resistance; CPR: C-reactive protein; IL-13: Interleukin-13.

**Table 3:** Summary of human studies evaluating the association between coffee consumption and liver diseases and MASLD.

Study Design	Population	Coffee Dose	Finding	Reference
Cohort with 12.4-year follow up	170,044 participants	>3.5 cups/day	Lowest risk of chronic liver disease and liver-related events.	[36]
Cross-sectional	8062 participants	>4 cups/day	21% lower NAFLD risk. Stronger association in women and in individuals with lower educational attainment.	[37]
Cohort	1079 participants	4–6 cups/day	59.3% reduced risk of MASLD.	[38]
Cohort 10.49-year follow up	185,437 participants	>2.5 cups/day	Lower risk of MASLD.	[39]
Longitudinal one-year follow up	6592 participants	≥2 cups/day	Lower risk of fibrosis progression in those with pre-existing MASLD.	[40]
Meta-analysis	897 participants (14 randomized controlled trials)	Highest vs. lowest coffee intake (study-specific categories)	No effect of coffee consumption on liver enzymes. Increase adiponectin concentration.	[41]

NAFLD: Non-alcoholic fatty liver disease; MASLD: Metabolic dysfunction-associated steatotic liver disease.

**Table 4:** Summary of human studies evaluating the association between coffee consumption and cardiovascular disease.

Study Design	Population	Coffee Dose	Finding	Reference
Cohort 12-5-year follow up	449,563 participants	2–3 cups/day (decaffeinated and instant coffee). 4–5 cups/day (ground coffee)	Lowest risk of incident CVD. Lower risk of incident arrhythmia.	[42]
Prospective cohort study	9964 participants never-smokers	2–4 cups/day	Lower risk of total CVD, coronary heart disease, myocardial infarction, and heart failure.	[43]
Meta-analysis	32 cohort studies	Moderate coffee consumption (middle category of each study: tertile 2, quartile 3, quintile 3, or sextile 3).	Men: Increased risk of CHD. Women: Reduced risk of CHD.	[44]
Meta-analysis	82,270 participants with T2D	4 cups/day	Lower risk of cardiovascular mortality, CHD mortality, CHD, and total cardiovascular events.	[45]
Cohort 13-years follow up	10,639 participants	≥540 g of coffee/day	Reduced risk of CVD mortality.	[46]
Prospective cohort	11,697 women	0, <1, 1, 2–3, and ≥4 cups/day (filtered caffeinated coffee)	No association and cardiovascular mortality.	[47]

T2D: Type 2 diabetes; CVD: Cardiovascular disease; CHD: Coronary heart disease.

**Table 5:** Summary of human studies evaluating the association between coffee consumption and gut microbiota modulation.

Study Design	Population	Coffee Dose	Finding	Reference
Clinical trial	16 participants	3 cups/day	Higher abundance of beneficial microorganisms (*Bifidobacterium* spp.).	[49]
Multi-cohort observational	22,867 participants from the U.S. and UK cohorts; validated in 211 additional cohorts (total validation n = 54,198)	Habitual coffee consumption (self-reported)	Associated with increased abundance and prevalence of *L. asaccharolyticus.*	[51]

**Table 6:** Summary of human studies and animal experimental evaluating the association between coffee consumption and coffee-derived bioactive compounds under fructose-related conditions.

Study Design	Population	Coffee Dose	Finding	Reference
Randomized crossover trial	10 participants	4 cups/day	Reduce hepatic glucose production.	[57]
Experimental	Rats fed a high-fat and high-fructose diet	1.18 g of ground medium-roast coffee for 14 weeks (human equivalent to 4 cups of coffee per day)	Reduce body weight, TG, and plasma glucose, and attenuating hepatic steatosis and IR.	[58]
Experimental	Rats fed a high-fructose diet	213 mg/kg body weight/day of coffee for 6 weeks (human equivalent to 3 cups per day). 284 mg/kg body weight/day of coffee for 6 weeks (human equivalent to 4 cups per day).	Low doses: Reduced body weight, fasting glucose, and uric acid levels. High doses: Additionally, decreased fasting TG and LDL-C concentrations.	[59]
Experimental	Rats fed a high-fructose diet	100 mg/kg/day for 4 weeks (equivalent to a human dose of 16.2 mg/kg/day)	Prevented adipose tissue accumulation and ameliorated hyperglycemia, hyperinsulinemia, hyperlipidemia, hepatic steatosis, and oxidative stress.	[60]
Experimental	Rats fed a high-fat/high-fructose diet	7.4 mL of coffee for 16 weeks (human equivalent to 75 mL/day in humans)	Reduction in NF-κB levels: Very dark roast: ~75% Dark roast: ~50% Unroasted coffee: ~30%	[61]
Experimental	Rats fed a high-fructose diet	Green or roasted coffee extract (200 mg/kg/day; human equivalent to 32.4 mg/kg/day) or chlorogenic acid (30 mg/kg/day; human equivalent to 4.86 mg/kg/day) for 12 weeks.	Green coffee, particularly combined with green tea, improved obesity-related metabolic, oxidative stress, and inflammatory outcomes; chlorogenic acid was more effective than caffeine.	[62]
Experimental	Rats fed a high-carbohydrate, high-fat diet with fructose	5% aqueous Colombian coffee extract (~3 g coffee/kg; human equivalent to 35 g coffee/Kg).	Improved glucose tolerance, reduced plasma insulin and systolic blood pressure, and attenuated cardiovascular and hepatic inflammation, fibrosis, and liver injury markers.	[63]
Randomized crossover trial	10 participants	4 cups/day	Reduce hepatic glucose production.	[57]
Experimental	Rats fed a high-fat and high-fructose diet	1.18 g of ground medium-roast coffee for 14 weeks (human equivalent to 4 cups of coffee per day)	Reduce body weight, TG, and plasma glucose, and attenuating hepatic steatosis and IR.	[58]
Experimental	Rats fed a high-fructose diet	213 mg/kg body weight/day of coffee for 6 weeks (human equivalent to 3 cups per day). 284 mg/kg body weight/day of coffee for 6 weeks (human equivalent to 4 cups per day).	Low doses: Reduced body weight, fasting glucose, and uric acid levels. High doses: Additionally, decreased fasting TG and LDL-C concentrations.	[59]

TG: Triglycerides; IR: Insulin resistance; LDL-C: Low-density lipoprotein cholesterol; NF-κB: Nuclear factor kappa B.

## Data Availability

No new data were created or analyzed in this study. Data sharing is not applicable to this article.

## Abbreviations

The following abbreviations are used in this manuscript:

AGEs	Advanced glycation end products
Akt	Protein kinase B
ALP	Alkaline phosphatase
ALT	Alanine aminotransferase
BMI	Body mass index
CGA	Chlorogenic acid
CHD	Coronary heart disease
CVD	Cardiovascular disease
DBP	Diastolic blood pressure
FFA	Free fatty acids
GPx	Glutathione peroxidase
IKK	IκB kinase
IL-1β	Interleukin-1 beta
IL-6	Interleukin-6
IL-8	Interleukin-8
IL-13	Interleukin-13
IR	Insulin resistance
IRS1	Insulin receptor substrate 1
LDL-C	Low-density lipoprotein cholesterol
MASLD	Metabolic dysfunction-associated steatotic liver disease
NADPH	Nicotinamide adenine dinucleotide phosphate
NAFLD	Non-alcoholic fatty liver disease
NF-κB	Nuclear factor kappa B
nNOS	Neuronal nitric oxide synthase
NO	Nitric oxide
PI3K	Phosphoinositide 3-kinase
RAGE	Receptor for advanced glycation end products
ROS	Reactive oxygen species
S1P	Sphingosine-1-phosphate
S1PR1	Sphingosine-1-phosphate receptor 1
S1PR3	Sphingosine-1-phosphate receptor 3
SOD	Superoxide dismutase
SphK1	Sphingosine kinase 1
SSB	Sugar-sweetened beverage
T2D	Type 2 diabetes
TG	Triglycerides
TIRAP	Toll/interleukin-1 receptor domain-containing adaptor protein
TLR4	Toll-like receptor 4
TNF-α	Tumor necrosis factor alpha
